# XB_2_Bi_2_ (X = Si, Ge, Sn, Pb): Penta-Atomic Planar Tetracoordinate Si/Ge/Sn/Pb Clusters with 20 Valence Electrons

**DOI:** 10.3390/ijms25052819

**Published:** 2024-02-29

**Authors:** Yan-Xia Jin, Jin-Chang Guo

**Affiliations:** Key Laboratory of Materials for Energy Conversion and Storage of Shanxi Province, Institute of Molecular Science, Shanxi University, Taiyuan 030006, China; jin__yx@163.com

**Keywords:** planar tetracoordinate silicon, planar tetracoordinate germanium, 20 valence electrons, double π/σ aromaticity

## Abstract

Planar tetracoordinate silicon, germanium, tin, and lead (ptSi/Ge/Sn/Pb) species are scarce and exotic. Here, we report a series of penta-atomic ptSi/Ge/Sn/Pb XB_2_Bi_2_ (X = Si, Ge, Sn, Pb) clusters with 20 valence electrons (VEs). Ternary XB_2_Bi_2_ (X = Si, Ge, Sn, Pb) clusters possess beautiful fan-shaped structures, with a Bi–B–B–Bi chain surrounding the central X core. The unbiased density functional theory (DFT) searches and high-level CCSD(T) calculations reveal that these ptSi/Ge/Sn/Pb species are the global minima on their potential energy surfaces. Born–Oppenheimer molecular dynamics (BOMD) simulations indicate that XB_2_Bi_2_ (X = Si, Ge, Sn, Pb) clusters are robust. Bonding analyses indicate that 20 VEs are perfect for the ptX XB_2_Bi_2_ (X = Si, Ge, Sn, Pb): two lone pairs of Bi atoms; one 5c–2e π, and three σ bonds (two Bi–X 2c–2e and one B–X–B 3c–2e bonds) between the ligands and X atom; three 2c–2e σ bonds and one delocalized 4c–2e π bond between the ligands. The ptSi/Ge/Sn/Pb XB_2_Bi_2_ (X = Si, Ge, Sn, Pb) clusters possess 2π/2σ double aromaticity, according to the (4*n* + 2) Hückel rule.

## 1. Introduction

The classical tetrahedral carbon (thC) concept was proposed independently by van’t Hoff and Le Bell in 1874, which formed the cornerstone of the development of organic chemistry [1,2]. In 1968, a hypothetical planar tetracoordinate carbon (ptC) structure was proposed by Monkhorst as a transition state structure to describe the isomerization of a chiral molecule with a four-coordinate asymmetric carbon atom [3]. Compared to the tetrahedral configuration, small bond angles lead to a larger repulsion between the ligands in the planar tetracoordinate carbon (ptC) configuration. Thus, the ptC systems are usually unstable relative to their thC counterparts. How can we stabilize a ptC system? Hoffmann, Alder, and Wilcox proposed the “electronic strategy” for stabilizing ptC species in 1970, based on analyzing the molecular orbital (MO) sequence of a planar methane system [4]. The first representative ptC molecule, 1,1-dilithiocyclopropane, was theoretically predicted by Collins and coworkers in 1976 [5]. Their innovative design ideas have provided us with inspiration and confidence in exploring planar hypercoordinate molecules. Since then, many ptC, planar pentacoordinate carbon (ppC), and planar hexacoordinate carbon (p6C) species have been designed theoretically, characterized by gas spectroscopy, and synthesized experimentally [6,7,8,9,10,11,12,13,14,15]. It should be noted that several aluminum-based ptC clusters, including Al_4_C^−^, Al_4_C^2−^, CAl_3_Si^−^, CAl_3_Ge^−^, Al_4_CH^−^, Al_11_C^−^, and Al_5_C_5_^−^, were experimentally detected in the gas phase via photoelectron spectroscopy (PES) [16,17,18,19,20]. Interestingly, the double-layered Al_11_C^−^ cluster possesses the exotic dynamic fluxionality, which can be regarded as the first aluminum-based ptC molecular rotor [21]. Interestingly, the concept of ptC can be further extended to the planar pentacoordinate carbon (ppC) and planar hexacoordinate carbon (p6C) species [22,23,24].

The peculiar structures of ptC, ppC, and p6C clusters have aroused great interest among chemists, while less attention has been paid to their silicon and germanium analogues beyond the traditional tetrahedron concept. Silicon, germanium, tin, and lead atoms lie directly under carbon in the same column of the periodic table and have some similarities in bonding properties as congeners. However, silicon, germanium, tin, and lead atoms, with their larger radii than carbon, have more stringent requirements for the coordination environment. Unexpectedly, in 2000, the first ptSi/Ge MAl_4_^−^ (M = Si, Ge) clusters were detected in a gas phase photoelectron spectroscopy (PES) experiment by Wang, which possess the fan-shaped *C*_2*v*_ structures [25]. Subsequently, theoretical researchers carried out a series of theoretical explorations and obtained some local minima systems containing the planar hypercoordinate Si (phSi), such as the *C*_2*v*_ B_n_E_2_Si series (E = CH, BH, or Si; *n* = 2–5), B_8_Si, C_58_Si, M_5_H_5_Si (M = Ag, Au, Pd, Pt), and Cu_6_H_6_Si species [26,27,28,29,30]. From an experimental point of view, only the global minima structures on the potential energy surface are most likely to be characterized. Opportunities and challenges coexist in designing and characterizing new forms of ptSi species in chemistry. The ptSi Si(CO)_4_ (*D*_2*h*_) cluster was reported as the most stable species by Belanzoni in 2006 [31]. ptSi/Ge M_4_Cl_4_X (M = Ni, Pd, Pt; X = Si, Ge), X_3_M_3_ (M = Cu, Li; X = Si, Ge), and XBe_4_H_5_^−^ (X = Si, Ge) as the GMs were predicted by our group [32,33,34]. It is worth mentioning that ppSi XMg_4_Y^−^ (X = Si, Ge; Y = In, Tl), SiMg_3_In_2_, and p6Si SiSb_3_M_3_^+^ (M = Ca, Sr, Ba) as the GM species were predicted, which further stimulated the enthusiasm of theoretical researchers to design phSi systems [35,36]. The phSi molecules have the unique structures, properties, and can serve as high-quality precursors for the design and synthesis of new materials, which can be applied in the fields of nanotechnology, catalysis, and optoelectronics. Based on theoretical calculations, we can predict a series of physical and chemical properties of silicon-containing species, providing theoretical guidance for the design of new silicon-containing complexes and materials. Recently, a series of breakthroughs have been made in the experimental synthesis and characterization of ptSi compounds [37,38,39,40].

How to design the stable ptSi clusters is still an open question to date. Here, we focus on penta-atomic clusters containing ptSis. In 2012, Alexandrova theoretically predicted the ptSi GM SiIn_4_^2−^ with 18 valence electrons (VEs), inspired by the isoelectronic ptC CAl_4_^2−^ cluster [41]. Two years later, Xu reported nine penta-atomic ptSi GMs with 14 VEs, including Li_3_SiAs^2−^, HSiY_3_ (Y = Al/Ga), Ca_3_SiAl^−^, Mg_4_Si_2_^−^, C_2_LiSi, and Si_3_Y_2_ (Y = Li/Na/K) [42]. Recently, the ptX XB_2_Be_2_ (X = Si, Ge, Sn, Pb) clusters were designed, each of which has 14 VEs [43]. In 2014, a series of ptX GMs, C_2_Si_2_X^q^ (X = C, Si, Ge, Sn, Pb; q = +1, 0, −1), with 19/20/21 VEs, were predicted by Ding [44]. We found that the Si–X distances seem to be conspicuously long in the C_2_Si_2_X (X = C, Si, Ge, Sn, Pb) clusters, due to the rigid Si–C–C–Si ligand chain. Can we improve this situation and design the more ideal ptSi/Ge/Sn/Pb systems? The answer is yes. 

The proper polarization of ligands is an effective strategy. Based on the fan-shaped structure of SiAl_4_, we replace two Al atoms on the top with the B atoms. As we all know, the boron atom has the same number of valence electrons as the aluminum atom, and it is more electronegative than aluminum. The isoelectronic substitution strategy is to replace the atoms in the system with one or more isoelectronic atoms, which is an effective way to develop new species. Using two N atoms to replace the remaining Al atoms in SiB_2_Al_2_, the ptSi structure is unable to be maintained. The ptSi SiB_2_P_2_, SiB_2_As_2_, and SiB_2_Sb_2_ clusters are all true minima, but their energies differ very little from those of the corresponding second structures. After many calculation attempts, we finally choose to use boron and bismuth atoms as the ligands. Based on the isoelectronic principle, using the flexible Bi−B−B−Bi ligand chain to stabilize the central X (X = Si, Ge, Sn, Pb) atoms, we theoretically predict the ptX XB_2_Bi_2_ (X = Si, Ge, Sn, Pb) in the current work. These 20 VEs ptSi/Ge/Sn/Pb clusters possess good thermodynamic and kinetic stability, although the Bi ligand atom has one lone pair (LP). Interestingly, these fan-shaped ptSi/Ge/Sn/Pb species have a unique 2π/2σ double aromaticity. These novel penta-atomic ptSi/Ge/Sn/Pb species not only enrich the family of planar hypercoordinate atoms, but also offer new ideas for designing unclassical molecules.

## 2. Results and Discussion

### 2.1. Structure and Stabilities

**Figure 1:**
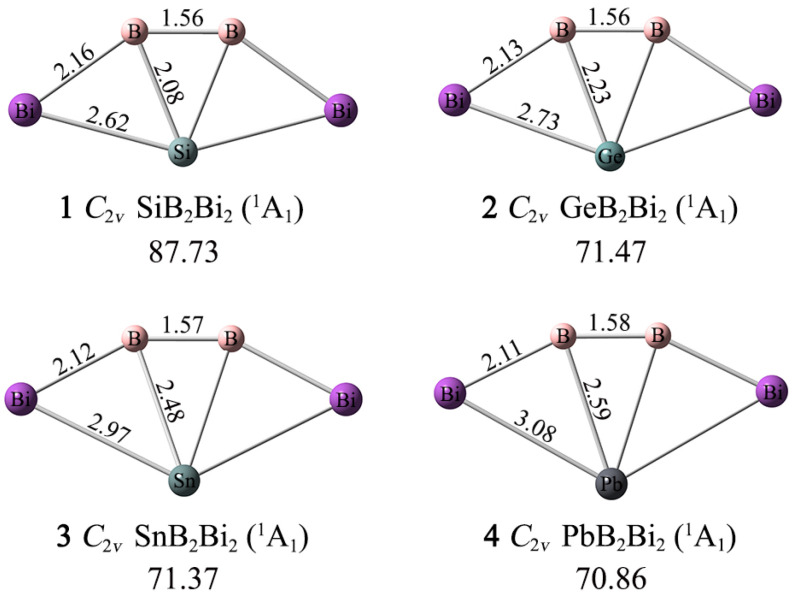
Optimized GM structures of XB_2_Bi_2_ (X = Si, Ge, Sn, Pb) clusters with bond distances (in Å) at the PBE0-D3(BJ)/def2-TZVP level. The lowest vibrational frequencies are shown (in cm^−1^).

As depicted in Figure 1, the XB_2_Bi_2_ (X = Si, Ge, Sn, Pb) (**1**–**4**) clusters assume perfectly planar fan-shaped geometries with *C*_2*v*_ (^1^A_1_) symmetry, whose X center is tetracoordinated in plane by two boron and two bismuth atoms. The ptSi/Ge/Sn/Pb **1**–**4** clusters are true GM structures on their potential energy surfaces, according to the unbiased computational global searches. For such penta-atomic clusters, the CK program can accurately achieve their global minima structures.

As shown in Figure 1, the lowest vibrational frequencies of **1**–**4** are 87.73, 71.47, 71.37, and 70.86 cm^−1^ at the PBE0-D3(BJ)/def2-TZVP level, respectively. In order to check for validity and reliability in terms of structures and the minima, the structure optimization and frequency analyses are also carried out for **1**–**4** using the second-order Møller–Plesset perturbation theory (MP2) with the def2-TZVP basis set [45]. As shown in Figure S1, the MP2 procedure produced essentially the same structures for **1**–**4** as the PBE0-D3(BJ) level, and only for minor bond distance differences. Thus, only PBE0-D3 and single-point CCSD(T)//PBE0-D3 data are to be discussed in the paper.

The Bi–B–B–Bi ligand chains are relatively rigid, and the B–B, B–Bi bond distances change little as the X atom changes. Specifically, the B–B bond distances grow slightly (1.56–1.58 Å) from **1** to **4**, while the B–Bi bond distances shrink slightly (2.16–2.11 Å). According to the covalent radii data recommended by Pyykkö, both the B–B and B–Bi links are close to a double bond (1.56 and 2.19 Å). The X–B, X–Bi bond distances become longer as the X atom grows from Si to Pb. The X–B bond distances (2.08/2.23/2.48/2.59 Å) are longer than the recommended covalent single bond lengths (2.01/2.06/2.25/2.29 Å), while the X–Bi bond distances (2.62/2.73/2.97/3.08 Å) are very close to the single bond lengths (2.67/2.72/2.91/2.95 Å). The bonding properties of the XB_2_Bi_2_ (X = Si, Ge, Sn, Pb) (**1**–**4**) clusters cannot be determined solely by the bond length data.

**Figure 2:**
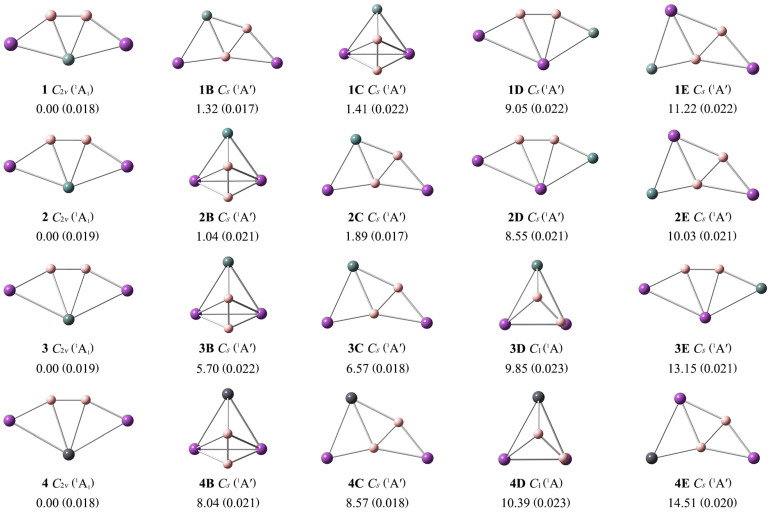
Optimized global minimum structures **1**–**4** of XB_2_Bi_2_ (X = Si, Ge, Sn, Pb) clusters and their four low-lying isomers (***n*B**–***n*E**) at the PBE0-D3(BJ)/def2-TZVP level. Relative energies are listed in kcal mol^−1^ at the single-point CCSD(T)/def2-TZVP//PBE0-D3(BJ)/def2-TZVP level, with zero-point energy (ZPE) corrections at PBE0-D3(BJ)/def2-TZVP. The T1 diagnostic values of the converged CCSD wavefunction are shown within parentheses.

To facilitate a comparison with the GM structures **1**–**4**, the lowest-lying isomeric ***n*B**–***n*E** structures are presented in Figure 2, along with the relative energies at the single-point CCSD(T)/def2-TZVP//PBE0-D3(BJ)/def2-TZVP level. Their optimized Cartesian coordinates are provided in Table S1. As shown in Figure 2, the T1 diagnostic values of the CCSD(T) method for clusters **1**–**4** are below the recommended threshold of 0.02. Thus, the single-reference method is reliable for the further electronic and structural analyses of these ptSi/Ge/Sn/Pb species. The ptX XB_2_Bi_2_ (X = Si, Ge, Sn, Pb) (**1**–**4**) are the true GMs, which are 1.32/1.04/5.70/8.04 more stable than the second low-lying isomers at the single-point CCSD(T) level.

Two boron atoms always exist as the B_2_ unit in isomers **1B**–**4E**, mainly due to the strong B–B covalent bonding. The isomer **1B** is formed by interchanging the positions of the boron and silicon atoms in the structure of GM **1**. In the periodic table, boron and silicon are diagonal elements, so they have small differences in electronegativity and geometric size. Thus, the interchange of their positions doesn’t make much difference to their energies. **1C** possesses a typical three-dimensional structure, while **1**/**1B**/**1D**/**1E** are all planar. **1D** is obtained by interchanging the Si atom with one Bi atom in structure **1**. If the Si atom in structure **1B** is exchanged with the adjacent Bi atom, **1E** is obtained. Similar associations are found between other isomers. Only a few three-dimensional geometric configurations appear, while the rest maintain the fan-shaped structures in Figure 2. Our global searches of the potential energy surfaces indicate that the GM ptX clusters **1**–**4** have a reasonably good thermodynamic stability (Figure 2).

**Figure 3:**
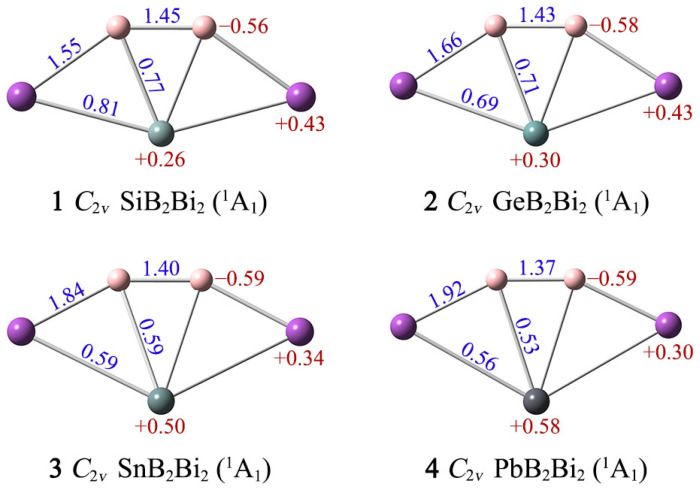
Calculated Wiberg bond indices (WBIs; blue) and natural atomic charges (in |e|; red color) of XB_2_Bi_2_ (X = Si, Ge, Sn, Pb) clusters.

Wiberg bond indices (WBIs) and natural atomic charges can help us understand more about the interactions between atoms in clusters **1**–**4**. As shown in Figure 3, the B–B/B–Bi links have WBIs of 1.45–1.37/1.55–1.92, indicating that they possess a certain double bond character. The corresponding X–B/X–Bi WBIs are 0.77–0.53/0.81–0.56, suggesting that they are close to covalent single bonds. In terms of natural atomic charges, the boron atoms carry partial negative charges −0.56/−0.58/−0.59/−0.59|e| in **1**/**2**/**3**/**4**, respectively, revealing that the changes in X have little effect on them. Both ptSi/Ge/Sn/Pb and Bi atoms carry partial positive charges in **1**–**4** (+0.26/+0.30/+0.50/+0.58|e| for X, +0.43/+0.43/+0.34/+0.30 |e| for Bi). The distribution of charge in the clusters is closely related to the electronegativity of the atoms. The degree of polarization of the X–B/X–Bi/B–Bi bonding in clusters **1**–**4** is not large, owing to small differences in the electronegativities of the elements (Si: 1.90; Ge: 2.01; Sn: 1.96; Pb: 2.33; B: 2.04; Bi: 2.02). Thus, the bonding between atoms in clusters **1**–**4** is dominated by covalent bonds.

Generally, the energy gap between the highest occupied molecular orbital (HOMO) and lowest unoccupied molecular orbital (LUMO) can reflect the chemical activity of the system. The greater the HOMO–LUMO gaps of one system, the weaker its chemical activity, and vice versa. As the ptSi/Ge/Sn/Pb GM species XB_2_Bi_2_ (X = Si, Ge, Sn, Pb) have sizable HOMO–LUMO gaps (2.79, 2.61, 2.52 and 2.46 eV), this suggests that these neutral ptSi/Ge/Sn/Pb species are electronically robust (see Figure S2). The ptC CB_2_Bi_2_ structure has a smaller HOMO–LUMO gap than those of XB_2_Bi_2_ (X = Si, Ge, Sn, Pb), suggesting its relatively weak stability. Indeed, the overall reactivity of one species is directly related to its absolute HOMO and LUMO energies. The energy of the HOMO usually reflects the tendency to be the electron donor of one system, whereas the energy of the LUMO corresponds to its electron acceptor properties. The HOMO energy increases as the X atom increases in XB_2_Bi_2_ (X = Si, Ge, Sn, Pb). Thus, SiB_2_Bi_2_ is most stable, while PbB_2_Bi_2_ is the easiest electron donor. The LUMO SiB_2_Bi_2_ is a typical π orbital whose energy is −2.86 eV. The LUMOs of XB_2_Bi_2_ (X = Ge, Sn, Pb) are σ-type orbitals, which are different to those of SiB_2_Bi_2_. The LUMO energy increases as X increases from Ge to Pb, although the changes are small. From the energy of LUMO, the activity of SiB_2_Bi_2_ is slightly larger than that of XB_2_Bi_2_ (X = Ge, Sn, Pb).

**Figure 4:**
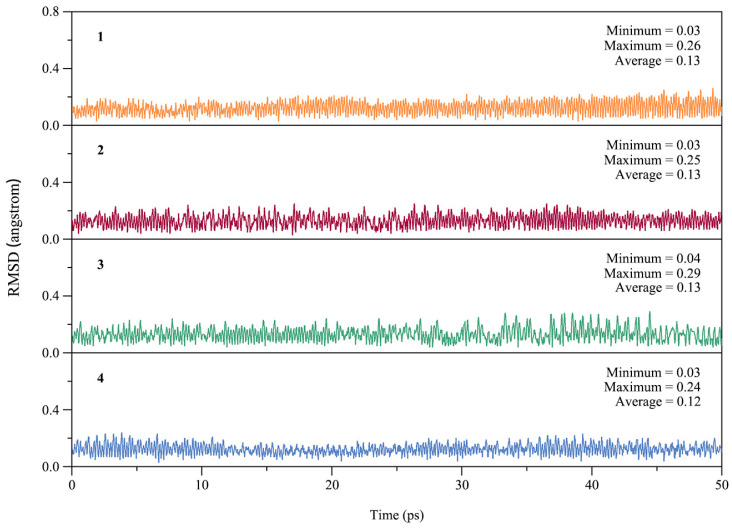
Calculated root-mean-square deviations (RMSDs) of GM clusters **1**–**4** of XB_2_Bi_2_ (X = Si, Ge, Sn, Pb) during the Born–Oppenheimer molecular dynamics (BOMD) simulations at 298 K.

From the point of view of experimental characterization, the dynamic stability of a cluster is as important as the thermodynamic stability. To assess the dynamic stability of GM clusters **1**–**4**, we performed Born–Oppenheimer molecular dynamics (BOMD) simulations at the PBE0/def2-SVP level. Each simulation was performed at near room temperature (298 K) for a time duration of 50 ps. The kinetic stability of **1**–**4** can be evaluated by examining the structural evolution during the BOMD simulations, as quantified by the root-mean-square deviations (RMSDs) in Figure 4. The average RMSDs 0.12–0.13 Å are relatively small for ptX clusters **1**–**4**, indicating good kinetic stability against isomerization or decomposition.

### 2.2. CMOs and AdNDP Analyses

**Figure 5:**
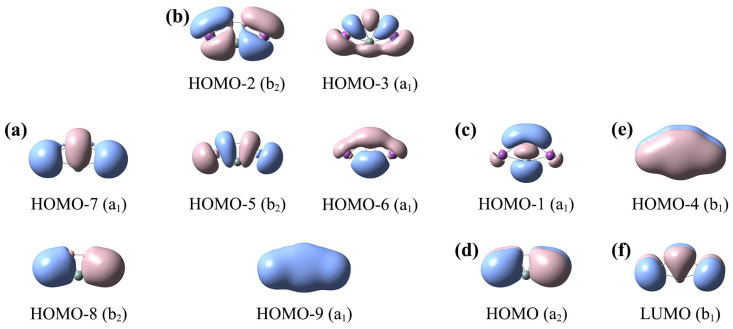
Analysis of canonical molecular orbitals (CMOs) of *C*_2*v*_ SiB_2_Bi_2_ (**1**) cluster. (**a**) Two Bi lone pairs (LPs); (**b**) the CMOs for Lewis-type two-center two-electron (2c–2e) B–B, B–Bi, Si–Bi σ bonds; (**c**) one σ CMO for delocalized B–Si–B 3c–2e bond; (**d**) one delocalized Bi–B–B–Bi 4c–2e π CMO; (**e**) one delocalized 5c–2e π CMO; (**f**) the π-type LUMO.

The canonical molecular orbital (CMO) analyses are important and fundamental, which can help us to understand the bonding characteristics of these ptX XB_2_Bi_2_ (X = Si, Ge, Sn, Pb) clusters. Since the electronic structures of clusters **1**–**4** are similar, we only analyze one representative SiB_2_Bi_2_ (**1**) cluster. SiB_2_Bi_2_ (**1**) has 20 valence electrons. Its ten occupied CMOs are depicted in Figure 5, which can be divided into five subsets based on the atomic orbital composition. The detailed compositions of the CMOs are shown in Table S2.

There are two CMOs (HOMO–7 and HOMO–8) in subset (a), which correspond to the lone pairs (LPs) of two Bi atoms. There are five σ CMOs in subset (b), which are responsible for five peripheral Lewis-type two-center two-electron (2c–2e) σ bonds along the five-membered ring. Subset (c) consists of only one CMO (HOMO–1), which is a delocalized σ framework. It is composed of 35% Si 3s/3p, 55% B 2s/2p, and 8% Bi 6p. Subset (d) includes one π-type CMO (HOMO), which corresponds to the delocalized π bond of the Bi–B–B–Bi ligands chain. The only CMO (HOMO–4) of subset (e) is a fully delocalized π CMO, including the contributions of 27% Si 3p, 46.4% B 2p, 25.5% Bi 6p. The delocalized π CMO (e) and σ CMO (c) cannot be transformed to Lewis-type π/σ bonds, indicating that *C*_2*v*_ SiB_2_Bi_2_ (**1**) possesses double (2π and 2σ) aromaticity according to the (4*n* + 2) Hückel rule. The double (2π and 2σ) aromaticity provides a strong guarantee for the stability of the SiB_2_Bi_2_ cluster. The LUMO (f) of SiB_2_Bi_2_ is a typical π orbital, endowing the system with a certain activity as an electron acceptor.

Natural Bond Orbitals (NBOs) are highly localized, and the NBO analysis method can generally search for one-center two-electron (1c–2e) bonds and two-center two-electron (2c–2e) bonds, transforming the system into a localized description in order to correspond with the Lewis formula. However, for multicenter systems with more than three centers, the NBO analysis method becomes overwhelmed. On the other hand, molecular orbitals (MOs) are highly delocalized, and the emergence of AdNDP serves as a transition between these two extreme forms of description. Since being proposed by Dmitry Yu. Zubarev and Alexander I. Boldyrev, AdNDP has been widely used as a tool to reveal the existence of delocalized bonds in specific systems, and it is a powerful tool for searching for n-center two-electron (*n*c–2e) bonds (*n* is less than the number of atoms contained in the cluster). Specifically, AdNDP can perform multicenter bond searches by utilizing density matrix information, which involves transforming multicenter bonds into localized descriptions to handle the complexity and diversity of chemical bonds. Therefore, it can provide more accurate information about chemical bonds, allowing for a deeper understanding. The closer the occupation numbers (ONs) obtained are to the ideal ON = 2.00|e|, the more strongly the bonding mode analysis can be guaranteed.

In the AdNDP analysis process, the following basic principles should be followed: (1) the fewer the residual electrons, the better; (2) the closer the occupation numbers are to the ideal occupation number of 2.00|e|, the better; (3) the smaller the number of orbital centers, the better; (4) avoid duplicate occupation numbers as much as possible; (5) the AdNDP orbital distribution satisfies molecular symmetry. Although AdNDP partially satisfies people’s needs for the bonding analysis of certain molecules, it also has limitations. For example, there is no absolute rule in searching for n-center two-electron (*n*c–2e) bonds, which often requires empirical analysis to complete, leading to the analysis results being influenced by human factors to some extent. Therefore, when performing a bonding analysis of a system, we need to consider the AdNDP analysis results in combination with CMO and other bonding analysis methods to ensure the rationality of the corresponding analysis. 

**Figure 6:**
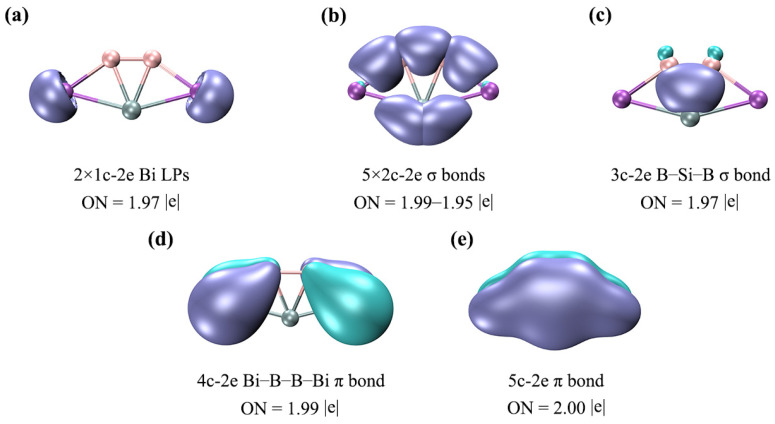
AdNDP bonding patterns of SiB_2_Bi_2_ (**1**). ONs are shown. (**a**) Two lone pairs (LPs) of two Bi atoms; (**b**) two-center two-electron (2c–2e) B–B, B–Bi, Si–Bi σ bonds; (**c**) one delocalized B–Si–B 3c–2e σ bond; (**d**) one delocalized Bi–B–B–Bi 4c–2e π bond; (**e**) one delocalized 5c–2e π bond.

To further elucidate the bonding nature of SiB_2_Bi_2_ (**1**), we carried out an AdNDP analysis and the results are given in Figure 6. As expected, the above bonding picture based on the CMO analysis is faithfully borne out from the AdNDP analysis. As shown in Figure 6a, the LPs of two Bi atoms have the occupation numbers (ONs) 1.97|e|, which are close to the ideal value (2.00|e|). In (b), there are one B–B, two B–Bi, and two Si–Bi 2c–2e σ bonds on the periphery, endowing the system with rigid boundaries. There is one delocalized B–Si–B 3c–2e σ bond in (c), which contributes the 2σ aromaticity. The 4c–2e delocalized π bond in (d) can give additional stability to the Bi–B–B–Bi ligand chain. Such a delocalized π bond between ligands is absent in the 16 VEs ptSi SiAl_4_ cluster. The fully delocalized π bond in (e) endows the ptSi system with 2π aromaticity. Electron delocalization is very beneficial for the stability of one planar cluster because the delocalized bonds can provide additional stabilization energy for the system. Here, the conclusion of the AdNDP is completely consistent with the above CMO analysis. It should be noted that there are two 2c–2e Si–Bi σ bonds, one delocalized 3c–2e σ bond, and one delocalized 5c–2e π bond, around the ptSi atom, which make the ptSi satisfy the stable eight-electron rule. Since the bonding of clusters **2**–**4** is similar to that of cluster **1**, we will not go into details here. Boldyrev and Simons have stressed that three σ bonds and one π bond around the C center are crucial for an 18-electron ptC cluster. This simple and effective judgment is also applicable to these ptSi/Ge/Sn/Pb XB_2_Bi_2_ (X = Si, Ge, Sn, Pb) systems. In other words, the eight-electron rule (three σ bonds and one π bond) is also applicable for ptSi/Ge/Sn/Pb species. As shown in Figure S3, the ELF_σ_ and ELF_π_ analyses indicate that there is double σ and π electronic delocalization in the SiB_2_Bi_2_ system. It should be noted that the existence of two π CMOs slightly weakens the aromaticity of the system, but the delocalization nature of the π electrons remains unchanged.

### 2.3. 2π + 2σ Aromaticity

**Figure 7:**
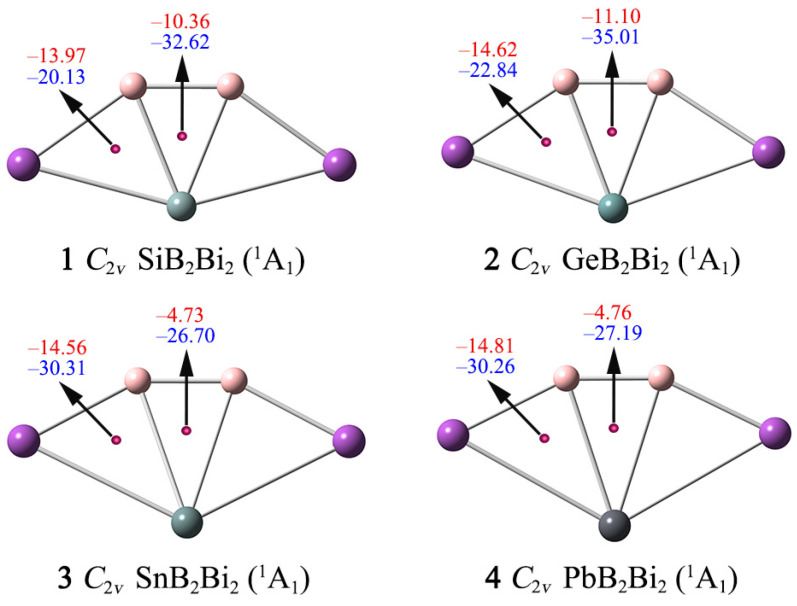
Nucleus-independent chemical shifts (NICSs) for clusters **1**–**4**. NICS(0), shown in blue, is calculated at the center of a triangle. NICS(1), shown in red, is calculated at 1 Å above the center of a triangle.

According to the CMO and AdNDP analyses, the ptSi/Ge/Sn/Pb clusters **1**–**4** are governed explicitly by double 2π/2σ aromaticity, as independently supported by their NICS data (Figure 7). As one of the main methods of aromaticity characterization, NICS can provide reliable quantitative analysis results of aromaticity. The probe (Bq) atoms are inserted above (0 and 1 Å) of the B–X–B (X = Si, Ge, Sn, Pb) triangle centers, and the magnetic shielding effect at these points is calculated. The negative NICS(0) and NICS(1) values can reflect σ and π aromaticity, respectively. In general, NICS values in the z direction (NICS_zz_) are more accurate than NICS values in evaluating the aromaticity. 

Here, we use the NICS(0)_zz_ and NICS(1)_zz_ data to assess the aromaticity of **1**–**4**. As shown in Figure 7, the NICS(0)_zz_ values at the geometric center of the B–X–B triangles are −32.62/−35.01/−26.70/−27.19 ppm for **1**–**4**, respectively, suggesting that the ptX XB_2_Bi_2_ (X = Si, Ge, Sn, Pb) clusters possess good σ aromaticity. The NICS(1)_zz_ values at 1 Å above the B–X–B, B–X–Bi triangles are all negative (from −4.73 to −14.81 ppm), indicating clusters **1**–**4** are π aromatic. Thus, the NICS(0)_zz_ and NICS(1)_zz_ data further support that systems **1**–**4** have double σ/π aromaticity, which is consistent with the conclusion obtained from the AdNDP analysis. 

**Figure 8:**
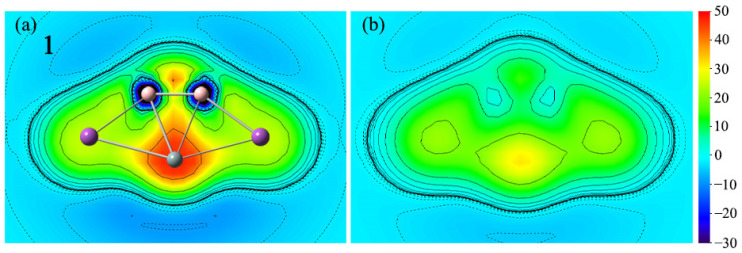
Color-filled maps of (**a**) ICSS(0)_zz_ and (**b**) ICSS(1)_zz_ (in ppm) for the SiB_2_Bi_2_ cluster (**1**). Positive values indicate aromaticity. The values 0 and 1 in parentheses represent the height above the molecular planes (in Å).

With only a few points of NICS_zz_ data, it does not seem enough. The magnetic criterion isochemical shielding surface (ICSS) calculation is handled in a three-dimensional grid of lattice points and direction and anisotropy effects can be quantified in a more straightforward way. In comparison with the NICS values at several certain points in the cluster, the research method of ICSS is more intuitive and vivid, which can comprehensively demonstrate the aromaticity of planar systems. To more intuitively observe the aromaticity, the color-filled maps of ICSS(0)_zz_ and ICSS(1)_zz_ of **1** are shown in Figure 8. Note here that positive ICSS_zz_ values indicate diatropic ring currents and aromaticity. As depicted in Figure 8, the positive ICSS(0)_zz_ and ICSS(1)_zz_ values reveal the corresponding aromaticity of **1**. The situation of **2**–**4** is similar, as shown in Figure S4.

### 2.4. Simulated IR Spectra

**Figure 9:**
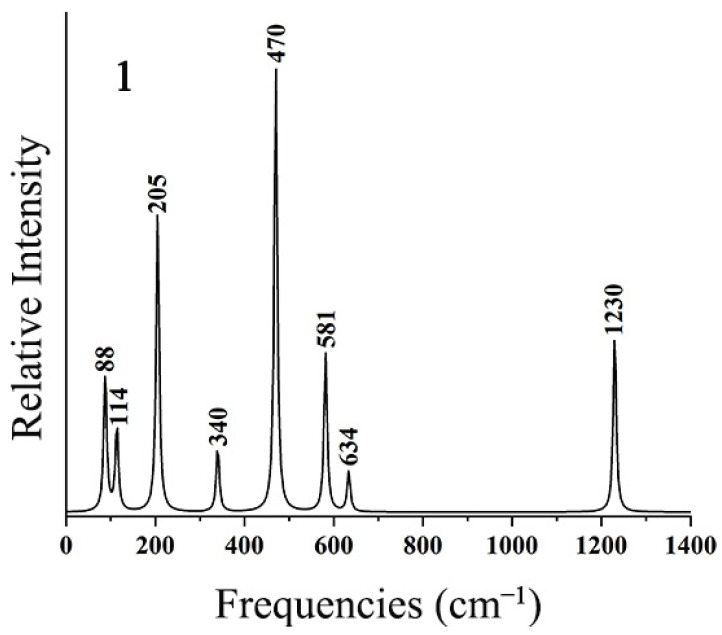
Simulated infrared spectra of SiB_2_Bi_2_ (**1**) at the PBE0-D3(BJ)/def2-TZVP level.

To facilitate further experimental characterization, the IR spectra of the ptX XB_2_Bi_2_ (X = Si, Ge, Sn, Pb) clusters were simulated at the theoretical level of PBE0-D3(BJ)/def2-TZVP. As shown in Figure 9, the strongest absorption peak at 470 cm^−1^ of SiB_2_Bi_2_ (**1**) mainly originates from B–Si anti-symmetry stretching vibration. The second strongest peak at 205 cm^−1^ originates from its anti-symmetry Si–Bi stretching vibration. The weak absorption peak at 1230 cm^−1^ corresponds to B–B stretching vibration. The other absorption peaks are mainly generated by coupled vibrations. As shown in Figure S5, the calculated IR spectra of **2**–**4** turned out to be similar to those of **1**. These simulated infrared spectra of the ptX XB_2_Bi_2_ (X = Si, Ge, Sn, Pb) clusters will provide a theoretical basis for subsequent experimental characterization.

## 3. Methods and Materials

The unbiased structural searches for XB_2_Bi_2_ (X = Si, Ge, Sn, Pb) clusters were performed at the PBE0/def2-SVP level, using the Coalescence Kick (CK) program [46,47,48]. More than 4000 stationary points (2000 singlets and 2000 triplets) were probed on the potential energy surfaces for each of the XB_2_Bi_2_ (X = Si, Ge, Sn, Pb) clusters. The top five low-lying isomer structures obtained in this way were then selected for re-optimization and frequency analyses at the PBE0-D3(BJ)/def2-TZVP level [49]. The higher-level single point calculations at CCSD(T)/def2-TZVP//PBE0-D3(BJ)/def2-TZVP were performed to obtain an accurate stability ordering of the top five isomers [50]. The ultimate relative energies of isomers were determined by the CCSD(T)/def2-TZVP energies plus the PBE0-D3(BJ)/def2-TZVP zero-point energy corrections.

Natural bond orbital (NBO) analyses were performed at the PBE0/def2-TZVP level [51]. Chemical bonding was elucidated via the canonical molecular orbital (CMO), adaptive natural density partitioning (AdNDP), and electron localization function (ELF) analyses [52,53]. The compositions of canonical molecular orbitals (CMOs) were analyzed using the Multiwfn progaram [54]. The Nucleus-independent chemical shifts (NICSs) were calculated to assess the aromatic characteristics of ptX clusters **1**–**4** [55]. The kinetic stability of the XB_2_Bi_2_ (X = Si, Ge, Sn, Pb) clusters were evaluated by Born–Oppenheimer molecular dynamics (BOMD) simulations at the PBE0/def2-SVP level of theory, at 298 K [56]. Isochemical shielding surface (ICSS) visualization was performed using the Multiwfn program [57]. All calculations for electric structures in this work were performed using the Gaussian 16 package [58]. 

## 4. Conclusions

We have computationally designed a series of ptSi/Ge/Sn/Pb XB_2_Bi_2_ (X = Si, Ge, Sn, Pb) clusters with 20 VEs in this study. These fan-shaped ptSi/Ge/Sn/Pb clusters are true global minima via computer structural searches and quantum chemical calculations. Chemical bonding analyses indicate that these ptX XB_2_Bi_2_ (X = Si, Ge, Sn, Pb) clusters have double 2π/2σ aromaticity, as well as LPs of two Bi, five Lewis-type σ single bonds, and one delocalized 4c–2e Bi–B–B–Bi π bond. Thus, the 20 VEs are ideal for these penta-atomic ptSi/Ge/Sn/Pb clusters. These penta-atomic ptSi/Ge/Sn/Pb species with novel and peculiar bonding will further enrich the study of planar tetracoordination compounds.

## Data Availability

Data is contained within the article and Supplementary Material.

## Supplementary Materials

The following supporting information can be downloaded at https://www.mdpi.com/article/10.3390/ijms25052819/s1.
