# Supplementary material for: XB2Bi2 (X = Si, Ge, Sn, Pb): Penta-Atomic Planar Tetracoordinate Si/Ge/Sn/Pb Clusters with 20 Valence Electrons
Source: Int J Mol Sci. 2024 Feb 29;25(5):2819. doi: 10.3390/ijms25052819 (PMC10931956; doi:10.3390/ijms25052819)

SUPPLEMENTARY INFORMATION

XB_2Bi_2 (X = Si, Ge, Sn, Pb): Penta-atomic Planar Tetracoordinate Si/Ge/Sn/Pb Clusters with 20 Valence Electrons

Yan-Xia Jin and Jin-Chang Guo*

Key Laboratory of Materials for Energy Conversion and Storage of Shanxi Province, Institute of Molecular Science, Shanxi University, Taiyuan, Shanxi 030006, China.

E-mail: guojc@sxu.edu.cn

Table of Contents

Table S1. Cartesian coordinates of optimized structures of the **1–4E** in the text.

Table S2. Orbital composition analysis of canonical molecular orbitals (CMOs) of the global-minimum structure **1** (C_{2v}, $^1A_1$) of SiB_2Bi_2 cluster.

Figure S1. Optimized GM structures of XB_2Bi_2 (X = Si, Ge, Sn, Pb) clusters with bond distances (in Å) at the MP2/def2-TZVP level. The lowest vibrational frequencies are shown (in cm^{-1}).

Figure S2. HOMO, LUMO, and HOMO–LUMO gap values of XB_2Bi_2 (X = Si, Ge, Sn, Pb) at the PBE0-D3(BJ)/def2-TZVP level. Those of ptC CB_2Bi_2 are also listed.

Figure S3. ELF_σ (a) and ELF_π (b) pictures of ptSi SiB_2Bi_2.

Figure S4. Color-filled maps of (a) $ICSS(0)_{zz}$ and (b) $ICSS(1)_{zz}$ (in ppm) for **2–4** clusters. Positive values indicate aromaticity. 0 and 1 in parentheses represent the height above the molecular planes (in Å).

Figure S5. Simulated infrared spectra of **2–4** clusters at the PBE0-D3(BJ)/def2-TZVP level.

Table S1. Cartesian coordinates of optimized structures of the **1–4E** in the text.

1 SiB_2Bi_2 (C_{2v},$^1A_1$)

Si	0.00000000	0.00000000	-0.69264364
B	0.00000000	0.78509201	1.24503740
B	0.00000000	-0.78509201	1.24503740
Bi	0.00000000	2.55212806	-0.01658652
Bi	0.00000000	-2.55212806	-0.01658652

1B (C_s,$^1A'$)

Si	-0.94882408	1.97728437	0.00000000
B	0.92192294	1.42269341	0.00000000
B	-0.04654403	0.08502439	0.00000000
Bi	2.34380697	-0.15531056	0.00000000
Bi	-2.23649803	-0.26903366	0.00000000

1C (C_s,$^1A'$)

Si	0.49035137	2.04064972	0.00000000
B	-1.70690907	0.06829469	0.00000000
B	-1.40945243	1.62902767	0.00000000
Bi	0.05251138	-0.22322716	1.51142293
Bi	0.05251138	-0.22322716	-1.51142293

1D (C_s,$^1A'$)

Si	-3.16275301	1.39061380	0.00000000
B	-1.31584304	1.82797793	0.00000000
B	0.25495699	1.47664304	0.00000000
Bi	-1.27884787	-0.53173907	0.00000000
Bi	1.87623308	0.09810415	0.00000000

1E (C_s,$^1A'$)

Si	1.43138892	2.25199596	0.00000000
B	0.04388196	0.89758792	0.00000000
B	-0.28310599	-0.72127209	0.00000000
Bi	-2.27725401	-0.05596116	0.00000000
Bi	2.05022600	-0.33451502	0.00000000

2 GeB_2Bi_2 (C_{2v},$^1A_1$)

Ge	0.00000000	0.00000000	-0.63270333
B	0.00000000	0.78509201	1.30497771
B	0.00000000	-0.78509201	1.30497771
Bi	0.00000000	2.55212806	0.04335379
Bi	0.00000000	-2.55212806	0.04335379

2B (C_s,$^1A'$)

Ge	0.18696613	1.90797657	0.00000000
B	-1.71776546	-0.34815627	0.00000000
B	-1.63805844	1.23866907	0.00000000
Bi	0.06503737	-0.39462539	1.51142293
Bi	0.06503737	-0.39462539	-1.51142293

2C (C_s,$^1A'$)

Ge	0.93697204	1.75591295	0.00000000
B	-0.94708287	1.28107631	0.00000000
B	-0.04008516	-0.08643272	0.00000000
Bi	-2.42327565	-0.22607460	0.00000000
Bi	2.12150137	-0.52287037	0.00000000

2D (C_s,$^1A'$)

Ge	-3.01359681	0.93725895	0.00000000
B	-1.22760024	1.57958290	0.00000000
B	0.37274913	1.40715661	0.00000000

Bi	−0.92542555	−0.76099895	0.00000000
Bi	2.13879125	0.21972204	0.00000000

2E (*C_s*,^{1}A′)

Ge	1.46438183	1.94856347	0.00000000
B	−0.02479150	0.70680685	0.00000000
B	−0.47737378	−0.88152502	0.00000000
Bi	−2.41338736	−0.06230811	0.00000000
Bi	1.87905734	−0.67842009	0.00000000

3 SnB_2Bi_2 (C_{2v},$^1A_1$)

Sn	0.00000000	0.00000000	1.24128239
B	0.00000000	0.79608423	−1.24018059
B	0.00000000	−0.79608423	−1.24018059
Bi	0.00000000	2.69689122	−0.29917056
Bi	0.00000000	−2.69689122	−0.29917056

3B (*C_s*,^{1}A′)

Sn	0.10781754	2.07980357	0.00000000
B	−1.65212270	−0.65341558	0.00000000
B	−1.90388277	0.91909968	0.00000000
Bi	0.07463344	−0.63444939	−1.51304437
Bi	0.07463344	−0.63444939	1.51304437

3C (*C_s*,^{1}A′)

Sn	1.29054355	1.89424475	0.00000000
B	−0.10998643	−0.17542506	0.00000000
B	−1.03243961	1.17202512	0.00000000
Bi	1.91643704	−1.00275401	0.00000000
Bi	−2.62505207	−0.19839343	0.00000000

3D (C_1,^{1}A)

Sn	1.86824000	−0.02450800	−0.06553900
B	−0.57684400	−0.53949200	1.94660500
B	0.46808700	0.62735800	1.67857000
Bi	−0.91073800	1.48914100	−0.07692100
Bi	−0.92844200	−1.47022100	−0.07671300

3E (C_s,^{1}A′)

Sn	3.15910817	−0.63956441	0.00000000
B	−0.54578919	−1.35498978	0.00000000
B	1.05753414	−1.42686307	0.00000000
Bi	−2.43143113	−0.33746760	0.00000000
Bi	0.49752590	0.89032887	0.00000000

4 PbB_2Bi_2 (C_{2v},$^1A_1$)

Pb	0.00000000	0.00000000	1.08732488
B	0.00000000	0.79608423	−1.39413810
B	0.00000000	−0.79608423	−1.39413810
Bi	0.00000000	2.69689122	−0.45312807
Bi	0.00000000	−2.69689122	−0.45312807

4B (C_s,^{1}A′)

Pb	0.06407749	1.82316412	0.00000000
B	−1.65007956	−0.93899848	0.00000000
B	−1.92800478	0.62910386	0.00000000
Bi	0.07612089	−0.89126497	−1.51304437
Bi	0.07612089	−0.89126497	1.51304437

4C (C_s,^{1}A′)

Pb	1.29928034	1.53072606	0.00000000
B	−0.31185493	−0.37957771	0.00000000

B	-1.08695072	1.05770040	0.00000000
Bi	1.61593793	-1.41614898	0.00000000
Bi	-2.81529889	-0.13698536	0.00000000

4D (C_1,^{1}A)

Pb	1.86824000	-0.02450800	-0.06553900
B	-0.57684400	-0.53949200	1.94660500
B	0.46808700	0.62735800	1.67857000
Bi	-0.91073800	1.48914100	-0.07692100
Bi	-0.92844200	-1.47022100	-0.07671300

4E (C_s,^{1}A′)

Pb	-1.73452637	-1.38043592	0.00000000
B	0.32297867	-0.35057301	0.00000000
B	1.12268273	1.08967496	0.00000000
Bi	-1.14601025	1.55419306	0.00000000
Bi	2.77255068	-0.23491311	0.00000000

Table S2. Orbital composition analysis of canonical molecular orbitals (CMOs) of the global-minimum structure **1** (C_{2v}, $^{1}A_{1}$) of SiB_2Bi_2 cluster.

CMO	Si (%)		B_2 (%)		Bi_2 (%)	
	s/p	total	s/p	total	s/p	total
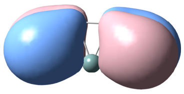 HOMO	0.00/0.00	0.00	0.00/13.87	13.87	0.00/**84.68**	**84.68**
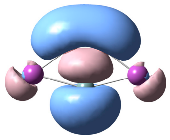 HOMO-1	9.53/**25.53**	**35.06**	9.30/**45.55**	**54.85**	0.00/7.72	7.72
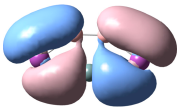 HOMO-2	0.00/**27.20**	**27.20**	4.72/**15.90**	**20.62**	0.00/**50.71**	**50.71**
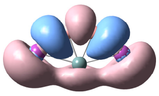 HOMO-3	1.87/5.58	7.45	0.00/**42.34**	**42.34**	5.29/**42.75**	**48.04**
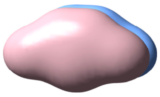 HOMO-4	0.00/**26.65**	**26.65**	0.00/**46.37**	**46.37**	0.00/**25.53**	**25.53**
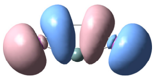 HOMO-5	0.00/3.33	3.33	**19.69**/11.82	**31.51**	**18.63/44.16**	**62.79**
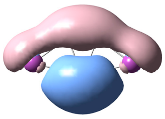 HOMO-6	**41.02**/4.76	**45.78**	**21.40**/13.64	**35.04**	3.58/13.32	**16.90**
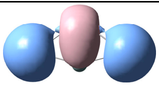 HOMO-7	2.65/3.01	5.66	**15.31/27.25**	**42.56**	**43.23**/2.93	**46.16**

CMO	Si (%)		B_2 (%)		Bi_2 (%)	
	s/p	total	s/p	total	s/p	total
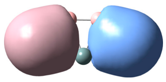 HOMO-8	0.00/9.34	9.34	6.37/13.29	**19.66**	**63.42**/0.00	**63.42**
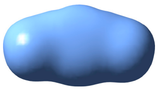 HOMO-9	**16.83**/3.36	**20.19**	**26.04**/8.36	**34.40**	**34.51**/3.32	**37.83**

Figure S1. Optimized GM structures of XB_2Bi_2 (X = Si, Ge, Sn, Pb) clusters with bond distances (in Å) at the MP2/def2-TZVP level. The lowest vibrational frequencies are shown (in cm^{-1}).

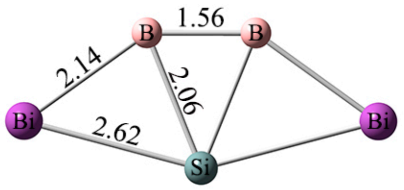

1 C_{2v} SiB_2Bi_2 ($^1A_1$)
86.5

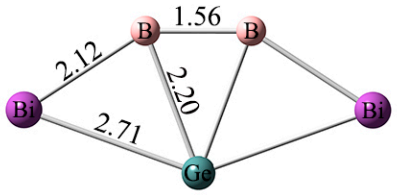

2 C_{2v} GeB_2Bi_2 ($^1A_1$)
69.7

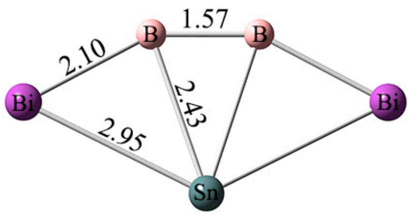

3 C_{2v} SnB_2Bi_2 ($^1A_1$)
71.0

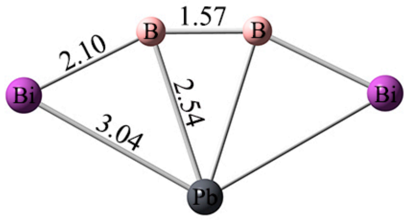

4 C_{2v} PbB_2Bi_2 ($^1A_1$)
71.9

Figure S2. HOMO, LUMO, and HOMO–LUMO gap values of XB_2Bi_2 (X = Si, Ge, Sn, Pb) at the PBE0-D3(BJ)/def2-TZVP level. Those of ptC CB_2Bi_2 are also listed.

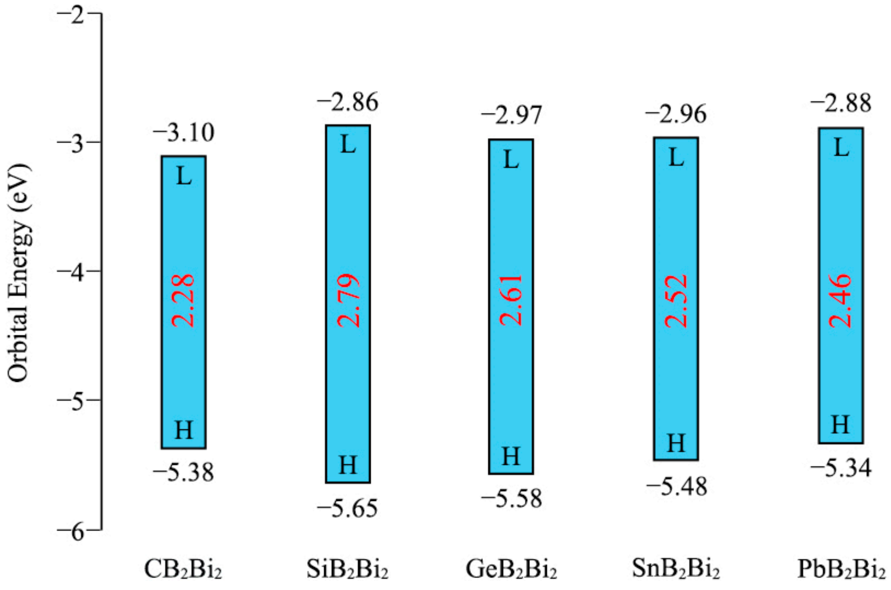

Figure S3. ELF$_σ$ (a) and ELF$_π$ (b) pictures of ptSi SiB_2Bi_2.

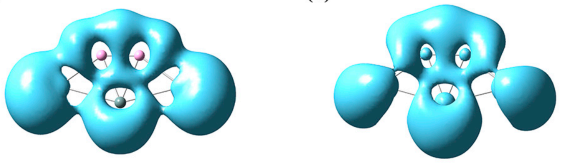

(a) ELF$_σ$ = 0.70

(b) ELF$_π$ = 0.54

1 C_{2v} SiB_2Bi_2 ($^1A_1$)

Figure S4. Color-filled maps of (a) ICSS(0)$_{zz}$ and (b) ICSS(1)$_{zz}$ (in ppm) for **2–4** clusters. Positive values indicate aromaticity. 0 and 1 in parentheses represent the height above the molecular planes (in Å).

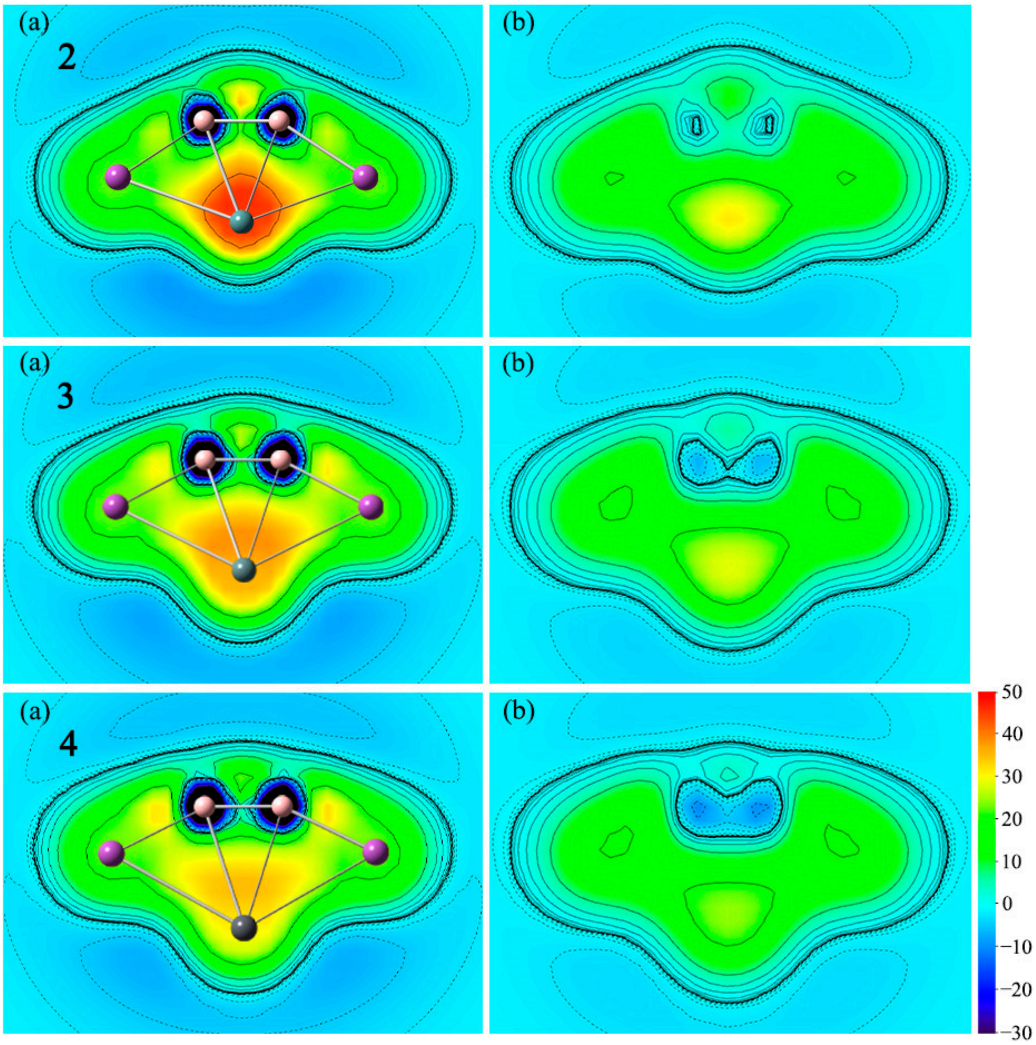

Figure S5. Simulated infrared spectra of **2–4** clusters at the PBE0-D3(BJ)/def2-TZVP level.

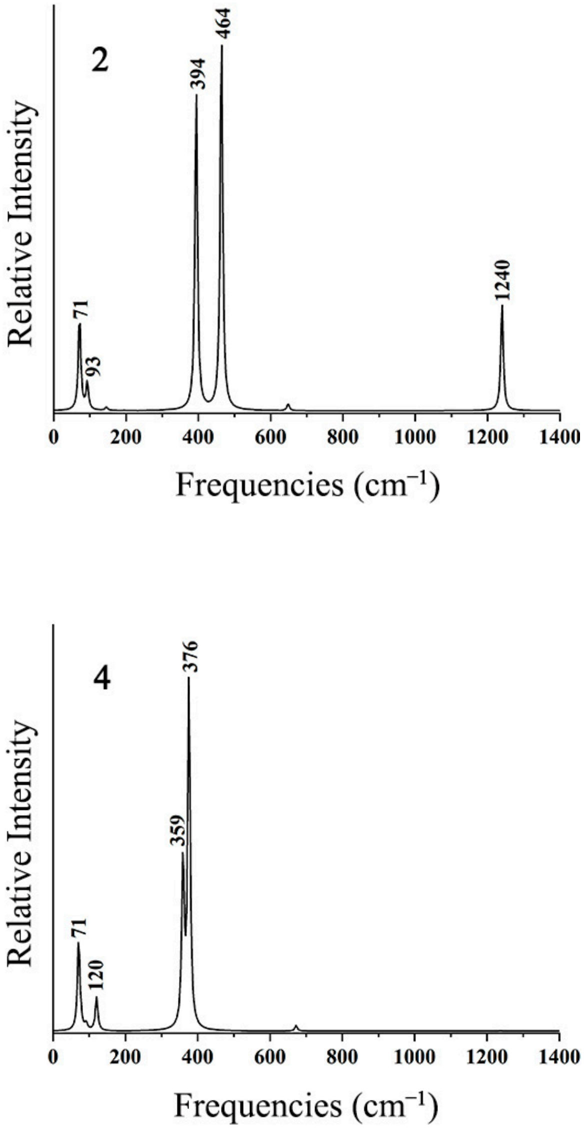

Supplement: Supplementary file 1 [file ijms-25-02819-s001.zip › ijms-2885778-supplementary.pdf]
